# Delivery strategy of mass annual single dose DEC administration to eliminate lymphatic filariasis in the urban areas of Pondicherry, South India: 5 years of experience

**DOI:** 10.1186/1475-2883-6-7

**Published:** 2007-08-24

**Authors:** B Nandha, C Sadanandane, P Jambulingam, PK Das

**Affiliations:** 1 Department of Health Economics, Vector Control Research Centre, Pondicherry, India; 2 Department of Vector Biology and Control, Vector Control Research Centre, Pondicherry, India

## Abstract

**Background:**

The recommended strategy for elimination of Lymphatic filariasis is single-dose, once-yearly mass treatment with anti-filarial drugs and the program is in operation on a national level in India. Rate of coverage and consumption is the most crucial factor in the success of Mass Drug Administration (MDA) program. In spite of massive efforts, the program demonstrated sub-optimal coverage and consumption in urban areas than rural. The involvement of Anganwadi workers (AWWs) of the Integrated Child Development Scheme (ICDS) as communicators and drug distributors was attempted to enhance the coverage and consumption in urban areas and the results presented here.

**Methods:**

An annual single dose MDA program was launched under the auspices of Freedom From Filariasis (FFF) program in Pondicherry, India, in the year 1997 and continued for five years. A questionnaire survey was carried out following all the treatment rounds (TRs) for assessing coverage of distribution and consumption Five percent of randomly selected households constituted the sample. All the members available in the selected household at the time of interview formed the respondent of the study.

**Results:**

The coverage of drug distribution during the TRs varied from 74.3 to 95.4 percent and consumption rate from 52.9 to 78.8. Among the respondents, 71% were aware of the MDA program and the source of information for 62.8% of them was through personal communication by the AWW. It was observed that 33.2% of the respondents who accepted the drug did so based on the trust on the AWW as a government representative. The main reason for non-consumption in all TRs was fear of side reaction (25.4 – 42.2%).

**Conclusion:**

The delivery-strategy of health information and Diethylcarbamazine (DEC) drug to the urban community using the AWWs could achieve relatively higher coverage and consumption than reported in other urban areas. In order to achieve the optimum level, it is imperative to equip the AWWs with current knowledge and skills, and design innovative Information, Education and Communication (IEC) campaign to target the less compliant groups. The beneficial effect of this delivery strategy may be used in similar urban settings to achieve the elimination of LF.

## Background

World wide 1.3 billion people are at risk of lymphatic filariasis (LF) infection and about 120 million people are affected in 83 countries [1]. Following the World Health Assembly resolution (WHA 50.29) on Elimination of LF as a public health problem by the year 2020, a global program (GPELF) was launched in 1999 to help endemic countries initiate national programs [2,3]. The global strategy to interrupt transmission of LF, is a once-yearly, single-dose, two-drug regimen (Albendazole with either Diethylcarbamazine (DEC) or Ivermectin) to be used by communities at risk with the goal of reaching 80% coverage for 4–6 years [4,5]. LF is an important public health problem in India where about 553.7 million people are at risk of infection in 243 districts [1], 29 million are parasite carriers and 22 million are with chronic disease, accounting for 40% of the global burden [6,7]. In accordance with global efforts several steps have been initiated towards elimination of LF in India and Mass Drug Administration (MDA) with DEC single dose is one of them. MDA with DEC was launched as a pilot project in 13 districts of 7 states in the year 1996 [8]. Subsequently, it has been expanded to target all endemic districts with introduction of albendazole in selected districts. The MDA campaign in 2005 covered a population of 463 million using DEC alone and 17.34 million with DEC plus albendazole combination [1]. A large scale trial on the feasibility and impact of co-administration of DEC plus albendazole in selected districts in the country was carried out during the year 2000 to 2005. The Indian Council of Medical Research (ICMR) Task Force has recommended this co-administration (DEC 6 mg/kg and albendazole 400 mg) strategy to all endemic districts and the government approval is awaited.

Previous studies have shown that urban areas recorded lower rates of coverage and compliance than rural areas [9,10]. Predictions indicate that at least 90% consumption is required to achieve the goal of elimination with five rounds of annual DEC based MDA and 11 rounds are required if the coverage is 60% [11]. Two important components in the MDA program are the delivery strategy of the drug for wider coverage and the communication strategy for compliance with the drug. In the year 1997 an annual single dose DEC mass treatment program was introduced under the auspices of Freedom From Filariasis (FFF) program in the urban endemic areas of Pondicherry and continued for five years. To achieve good coverage and consumption, Anganwadi workers (AWWs) of the Integrated Child Development Scheme (ICDS) who are close associates of the community were involved in planning and implementation of MDA and the program was evaluated independently. This paper reports the coverage of distribution and consumption of the drug in five rounds of annual single dose DEC administration in the urban areas of Pondicherry involving AWWs as motivators and drug distributors.

## Methods

### Study area

The study was conducted in Muthialpet zone of Pondicherry, South India, with a population of 47446, comprising of 8 administrative units or wards over a 9 sq km area. The population in these wards ranges from 4130 to 7438. Over-population, migration and unplanned construction in this zone have led to the creation of mosquito breeding sources. The area is endemic for filariasis with an infection rate of 3.82 in the year 1997 (VCRC Annual report, 1998) and has been under selective chemotherapy and routine vector control (anti-larval) operations. The health care facilities in this area include a Government General Hospital, a medical College hospital and many private clinics. Mass anti-filarial drugs were not administered to the study community prior to the implementation of MDA with DEC.

### Implementation strategy

Mass DEC distribution began in 1997 based on a house-to-house approach involving the AWWs. The job requirements of this female community health worker includes supplementary nutrition, immunization, health check ups, referral services, treatment of minor illness, nutrition and health education for women, preschool education for children aged 3–6 years, co-operation in improving supportive services such as water supply and sanitation and integrated services. An AWW is deputed for a population of 500 – 1000. They have frequent contact with the people in their respective areas and have gained acceptance as caretakers of the community. We utilized this strong community based resource as an existing, established infrastructure in the field of public health for popularizing the program and involving the community in the mass DEC program. Prior to each round of drug distribution, orientation and training sessions were conducted for the 69 AWWs who were involved in the MDA program. The main focus was on the disease cause, transmission, prevention and the necessity for mass annual DEC administration and methods and conditions of delivering the drug with possible side reactions and management. The AWWs in turn educated the community through interpersonal communication techniques during enumeration of eligible population prior to drug distribution. The feed back from the AWWs was received after every round and the program was modified accordingly. Campaigns through television, radio, newspaper, banner, pamphlets and posters were also conducted in a small scale. Door to door distribution of the drug was carried out with in a period of one week using a family enumeration register. Based on the requirement of each family, the DEC tablets were packed in separate covers and delivered to all the households. In case of absentees, the drug was left with available family member or neighbor. Children below two years, pregnant ladies and people under medication for chronic diseases were exempted from drug administration. A standard dose of DEC was worked out based on age, which corresponds to 6-mg/kg-body weight.

### Design of the study

Assuming that the expected consumption rate will be with in 60 ± 3%, with 95% confidence the minimum required sample size was arrived at 1100 respondents and the corresponding percent of population was 2.5%. Hence, 5% of the total households in the study area were selected using simple random technique. All the members in the selected household and available at home at the time of interview formed the respondent of the study. The purpose of research was communicated to the selected household members and their oral informed consent was obtained before administering the questionnaire. They were assured of confidentiality and anonymity.

The assessment in this study was made in terms of proportion of people who have actually received DEC tablets (= coverage of drug distribution), those who have consumed the tablet (= consumption of tablet out of sampled population) and compliance (= consumption of tablet out of those received tablets) in the selected areas. A household questionnaire survey was carried out with in fifteen days of drug distribution following all the treatment rounds (TRs), for assessing the coverage of distribution and consumption. A semi-structured questionnaire in the local language (Tamil) was used to collect information on coverage, consumption and side effects of the drug, family details of drug consumption, community's perception of drug distribution, etc. People were also asked about the channel through which they came to know about the drug distribution and which communication method had influenced them. During the final TR information was also collected about the number of times the respondents had received and consumed the drug during the previous rounds.

Database was organized using excel spreadsheet and data cleaning was carried out by verifying any inconsistency against the original questionnaire. Analysis of data was done using SPSS version.13. Mantel-Haenszel proportion test was carried out for significance.

## Results

### Feedback from AWWs

According to the feedback from AWWs the average number of households covered by a single worker in a day was 49 ± 32 and the number of individuals given tablets was 168 ± 105. To distribute the drugs, the average time taken was 5 ± 2 days with 3 ± 1 hours per day by each worker. Except a single case admitted in the hospital during the first round for giddiness, no other serious side reactions were reported to them. Those with mild to moderate side reactions were referred to the nearest Primary Health Centre. Majority of the AWWs (78.3%) felt that for achieving high consumption rate, drug distribution should be preceded by a strong Information, Education and Communication (IEC) through different media.

### Household survey

The total number of households visited and the total number of individuals interviewed after the treatment rounds (From 1997 to 2001) ranged from 263 to 689 and 1179 to 3195 respectively (Table 1). The same households could not be followed up during the entire period under study owing to the migratory nature of the urban population. Assessments made based on the residential status of the people showed that the residents in two of the 8 wards belonged to high income group and the rest of the wards had middle and lower income groups.

**Table 1:** Coverage of distribution and compliance of annual single dose DEC in Pondicherry during five consecutive rounds

	Rounds				
	
Item	TR 1	TR 2	TR 3	TR 4	TR 5
Sample	1179	3195	2976	3127	1953
No. received drug	1020	2373	2791	2983	1519
% received	86.5	74.3	93.8	95.4	77.8
No. consumed drug	850	1947	2346	2040	1034
% consumed	72.1	60.9	78.8	65.2	52.9
Compliance rate	83.3	82.0	84.1	68.4	68.1

### Coverage of DEC distribution and compliance

In all the TRs, the 8 wards included in the study had received DEC tablets and the proportion of respondents who had received the drug varied from 74.3 to 95.4%. Between the wards, estimates of mean coverage of individuals during the TRs varied from 45.9 to 95.0 percent. It was observed that the percentage of respondents who had directly received treatment from the drug distributors ranged from 20.7 to 44.4% during the TRs and was lowest in the fifth TR. A significantly higher (p =< 0.05) proportion of females (76.06%) received the drug in the fifth TR than males (67.28%) and no difference was observed in the other rounds.

**Figure 1:**
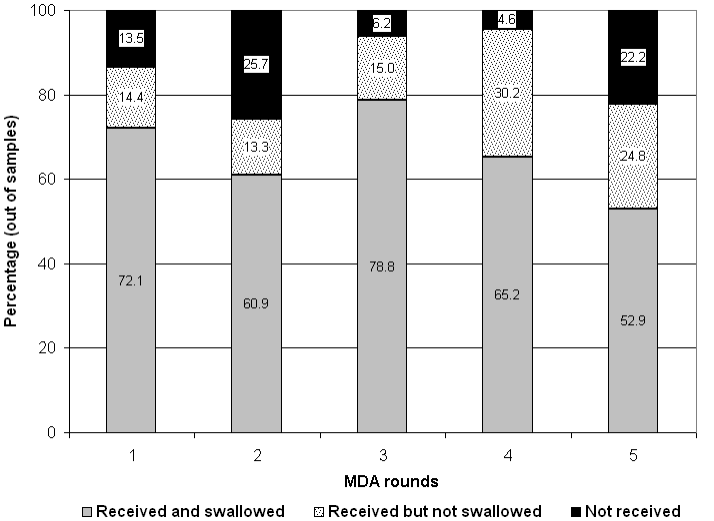
Coverage of DEC drug distribution and consumption in different rounds of treatment.

The average consumption rate for the study period was 66.1%. However the consumption rate varied in different TRs, showing a high rate of 78.8 in TR 3 and a low rate of 52.9 in TR 5 (Figure 1). Ward to ward variation was also observed, the consumption rate ranging from 37.1% to 92.0%. The variation seen in coverage of drug distribution and consumption was significantly different among different rounds and wards. However, with in the same TR, the relation between coverage and consumption was not found to be significant (R2 = 0.0055; Y = 0.0539x + 74.288). Treatment coverage and consumption was found to be lower in the two wards with high-income group residents in all TRs, being statistically significant in TR4 and TR5 (p =< 0.05).

The community compliance rate ranged from 68.1 to 83.3% in different TRs and 46.7 to 95.8% in different wards. The rate of supervised consumption among the TRs was in the range of 1.66 – 9.93%. The proportion of people who received the drug but did not consume ranged from 13.3 to 30.2% (Figure 1) in different TRs. The main reason for non-compliance in all TRs was fear of side reaction (25.4 – 42.2%). As many as 16.2 – 21.3% of respondents did not feel the necessity to adhere to treatment since they do not have disease symptoms and 15.6 – 21.2% were undergoing treatment for other ailments (Table 2). The low compliance rate in TR 4 was due to a death attributed to drug consumption in TR 4. Among those who consumed the drug, it was observed that the trust on the AWW as a government representative was the motive for 33.2% of the respondents and the fear of getting filariasis impelled 29.3% to consume. About 22% consumed as they were aware of its beneficial effect of the drug and 15.6% could not attribute any reason. Treatment compliance was observed to be significantly (p =< 0.05) lower in elderly respondents >61 years of age. Of those who were interviewed during the final treatment round 8.5% had consumed the drug in all the treatment rounds, while 23.2% had never complied with the treatment since the inception of the FFF program. Compliance with 2 rounds was the highest (32.2%) followed by three rounds (16.5%). Undergoing treatment for some ailment was the major reason for systematic non compliance. The proportion of males (47.6%) who did not consume the drug in any of the TRs was significantly (p =< 0.05) higher than that of females (20.3%). A significantly (p =< 0.05) higher proportion of females (10.3%) adhered to all the rounds of treatment than males (2.9%).

**Table 2:** Reason for not consuming DEC tablets among those received in different rounds of treatment

Reasons	TR 1 *n *= 170		TR 2 *n *= 426		TR 3 *n *= 445		TR 4 *n *= 943		TR 5 *n *= 485	
	
	No	%	No	%	No	%	No	%	No	%
Aged	12	7.1	13	3.1	11	2.5	21	2.2	15	3.1
Sick/under treatment	36	21.2	79	18.5	70	15.7	147	15.6	103	21.2
Pregnant and lactating	13	7.6	22	5.2	35	7.9	38	4.0	17	3.5
Fear of side reaction	47	27.6	120	28.2	133	29.9	398	42.2	123	25.4
Forgot	22	12.9	65	15.3	87	19.6	99	10.5	84	17.3
No disease/not willing	30	17.6	88	20.7	95	21.3	153	16.2	102	21.0
Out of station	10	5.9	39	9.2	14	3.1	87	9.2	41	8.5

The rate of adverse reactions observed ranged from 0.6 to 7.6% in different TRs, lowest being in TR 5 and highest in TR 1. This difference was statistically significant (p =< 0.05). Fever, headache and dizziness were the common adverse reactions reported. The average duration of any side reaction was half a day and did not prevent the respondents from carrying out their normal routine work.

About 71% of the respondents were aware of the drug distribution program. Personal communication by the AWW was the source of information for 62.8% of them. A total of 59.2% preferred to receive information on other health matters also through the AWWs. Other sources of information were advertisements in Television, Radio, news papers, banners, pamphlets and posters. Having seen Television advertisements was mentioned considerably more often by the women folk (27.6%) than men. Those who received information through print media was very low (7.7%) and was found to be gender dependent (59.3% men compared to 31.2% of women).

## Discussion

Massive efforts have been taken by the national and state governments along with World Health Organization (WHO), towards elimination of LF in India as a public health problem. In a country like India, annual MDA is an economic option [12] and the existing health care system is capable of operating the program [9,10]. However studies have shown that the main limitation in this program is a comparatively poor coverage of drug distribution and consumption in urban areas [9,10]. The rate of coverage and consumption is the most crucial factor in the success of MDA program and this is to a large extent dependant on the type of personnel involved in drug distribution. Programs based on community health workers have been successfully employed to address several health problems throughout the world [13-15]. The present study has demonstrated the role of AWWs in achieving more than 70% coverage of DEC drug distribution and more than 68% compliance in all the five TRs in urban areas. This is higher than the coverage of 53% [9] and 44.9% [10] and compliance of 35% [9] and 23.1% [10] reported earlier in Tamil Nadu and Orissa, India respectively. Due to periodic contact with the community members, the AWWs have earned the trust of their respective communities. This could be a significant contributory factor for the higher rate of coverage and consumption observed in the study. Enhanced compliance could have a major impact on the number of annual rounds of MDA required to achieve elimination. However, the coverage and consumption was significantly different in various TRs and between wards. The migratory nature of the population in urban areas is perhaps the reason for the difference observed. Fear of side reaction was the most common cause for non consumption of the drug. A high rate of side reaction in TR 1 affected the acceptance rate in TR 2. Following adequate mobilization with health education messages the acceptance rate went up again in TR 3. In different rounds of MDA 17.6–21.3% of the respondents did not adhere to treatment, due to the reason that they do not have disease symptoms or manifestation. One way of interpreting this is that side reactions and need for apparently healthy people to consume the tablets are not accepted by the community. This is suggestive of a low health literacy of the community, a factor that can have significant bearing on the individual's ability to comprehend the necessity of preventive care utilization [16]. Another reason for low awareness is inadequate publicity through media. To tackle the problem of systematic non-compliance reported during the final TR, it is essential to deliver appropriate health information designed specifically to address people's concerns and fears about the intervention.

The percentage of coverage and consumption was found to decline with increasing age as observed in another study [17]. Income level seemed to play an independent significant role, compliance being lowest among the high-income group. Feed back from the AWWs revealed that entering the premises of residents in high-income areas for drug distribution itself was difficult as the entrances were locked most of the time, and wherever entry was possible the response was poor. These people were not willing to take preventive medication and this could perhaps be due to the fact that for such people preventive medication is not a felt need as they could afford treatment from health facilities of their choice.

Throughout the study period, we depended on the AWWs for dissemination of information and it is evident from the findings that the message on DEC drug distribution had percolated into the community. But, the achieved coverage and consumption levels are not sufficient to completely interrupt transmission and low-level disease transmission and occurrence of new infections would continue. Therefore, from the elimination point of view, there is a need to enhance the existing levels of compliance with the AWWs as drug distributors. To achieve this, the enthusiasm of the AWWs should be maintained by motivational activities and they should be equipped with necessary knowledge and skills through effective re-training to target less compliant groups. Even though we attempted IEC through electronic and print media on a small scale, the results indicate that it was not adequate. Further efforts are necessary to harness the interests of target communities in order to enable passive beneficiaries to modify their behavior and change mind sets and transform into active accountable stakeholders in the MDA program. It is therefore imperative to have an innovative IEC that is appropriate to the local environment, health system, social structure, culture, population density and method of drug distribution[2]. Urban areas may benefit from education through social organizations such as neighborhood associations, housing associations, youth organizations etc. that foster social capital.

## Conclusion

This study shows that the strategy adopted for MDA in urban areas using AWWs has achieved enhanced coverage and consumption. Two conclusions can be drawn from the results obtained in this study. Firstly, MDA to urban population utilizing the services of AWWs as motivators and drug distributors is operationally feasible. Secondly, even though the improved coverage and consumption achieved are not sufficient to completely interrupt transmission, the levels have reached a stage which can be further enhanced to optimum levels with adequate mobilization through innovative IEC campaigns. This will pave way to our efforts to achieve the goal of elimination of LF as a public health problem.

## Competing interests

The author(s) declare that they have no competing interests.

## Authors' contributions

BN participated in the design of the study and fieldwork, performed data management and analysis and prepared the manuscript.

CS participated in training of AWWs and coordinated fieldwork

PJ planned and executed field work and edited the manuscript

PKD conceived the study, provided logistic support and edited the manuscript

All the authors read and approved the final manuscript
